# DNA metabarcoding of spiders, insects, and springtails for exploring potential linkage between above- and below-ground food webs

**DOI:** 10.1186/s40851-018-0088-9

**Published:** 2018-02-15

**Authors:** Hirokazu Toju, Yuki G. Baba

**Affiliations:** 1 Center for Ecological Research, Kyoto University, Hirano 2-509-3, Otsu, Shiga 520-2113 Japan; 2 Precursory Research for Embryonic Science and Technology (PRESTO), Japan Science and Technology Agency, Honcho 4-1-8, Kawaguchi, Saitama 332-0012 Japan; 3 Institute for Agro-Environmental Sciences, NARO, Kannondai 3-1-3, Tsukuba, Ibaraki 305-8604 Japan

**Keywords:** Above- and below-ground linkage, Collembola (springtails), DNA barcoding, Ecological communities, Food webs, Illumina sequencing, Insects, Networks, Molecular gut content analyses, Predator–prey interactions

## Abstract

**Background:**

Understanding feedback between above- and below-ground processes of biological communities is a key to the effective management of natural and agricultural ecosystems. However, as above- and below-ground food webs are often studied separately, our knowledge of material flow and community dynamics in terrestrial ecosystems remains limited.

**Results:**

We developed a high-throughput sequencing method for examining how spiders link above- and below-ground food webs as generalist predators. To overcome problems related to DNA-barcoding-based analyses of arthropod–arthropod interactions, we designed spider-specific blocking primers and Hexapoda-specific primers for the selective PCR amplification of Hexapoda prey sequences from spider samples. By applying the new DNA metabarcoding framework to spider samples collected in a temperate secondary forest in Japan, we explored the structure of a food web involving 15 spider species and various taxonomic groups of Hexapoda prey. These results support the hypothesis that multiple spider species in a community can prey on both above- and below-ground prey species, potentially coupling above- and below-ground food-web dynamics.

**Conclusions:**

The PCR primers and metabarcoding pipeline described in this study are expected to accelerate nuclear marker-based analyses of food webs, illuminating poorly understood trophic interactions in ecosystems.

**Electronic supplementary material:**

The online version of this article (10.1186/s40851-018-0088-9) contains supplementary material, which is available to authorized users.

## Background

Above- and below-ground biological communities are tightly linked with each other, collectively driving terrestrial ecosystem dynamics [1–3]. The above-ground parts of plants photosynthesize carbohydrates, of which 20% are directly supplied to below-ground mycorrhizal fungi in exchange for soil nutrients [4, 5]. The vast majority of carbohydrates then flow into soil food webs by being consumed by fungivorous invertebrates [e.g., springtails (Collembola)] [6, 7], which are subsequently preyed on by various groups of arthropod predators [8, 9]. As some groups of predators sustained by below-ground biomass also eat above-ground prey [10–12], the presence of such generalist predators has been expected to stabilize above-ground food webs through the regulation of population dynamics of herbivorous insects in natural and agricultural ecosystems [13, 14]. Although these insights highlight the importance of ecosystem services provided by generalist predators linking above- and below-ground communities, we are just starting to understand the structure of food webs linking both below- and above-ground invertebrates.

Spiders (Araneae) are among the most important groups of generalist predators potentially connecting above- and below-ground food webs [14, 15]. Spiders are known as major generalist predators stabilizing community dynamics of herbivorous insects, working as top-down regulators of above-ground food webs through trophic cascades [16]. As the body size and/or population size of spiders is limited by the amount of available prey biomass [11, 17], resource subsidies from below-ground food webs have been expected to strengthen the top-down regulation of above-ground food webs by spiders. In fact, pioneering studies have shown that springtails (Collembola) could be major “alternative prey” of web-weaving and wandering spiders, potentially sustaining populations of those spiders [8, 9, 11, 13, 18]. However, the number of spider species analyzed in these previous studies was low presumably due to difficulty in identifying many small spiders.

DNA barcoding techniques, which allow detection of prey DNA from predator samples, have recently been applied to studies of various types of predator–prey and parasite–host interactions [19–22], revolutionizing our understanding of species-rich food webs. Prey profile data based on high-throughput sequencing (e.g., 454, Illumina, and Ion-Torrent sequencing) have come to reveal poorly explored trophic interactions in the wild, providing novel insights into trophic interactions [23–26]. High-throughput sequencing analyses of spider diets have also indicated that spiders prey on both above- and below-ground arthropods [27, 28]. However, each of these studies focused on prey compositions of a single spider species. Thus, it remains a major challenge to identify diet composition of a spider community to determine above- and below-ground linkages. Once a high-throughput research workflow for investigating prey of multiple spider species is established, we will be able to discuss how spider communities drive terrestrial ecosystem processes in light of niche partitioning within spider functional guilds [29, 30].

In this study, we explored the structure of a food web involving multiple spider species and their Hexapoda prey by developing a new DNA metabarcoding approach. We first designed universal primers targeting broad taxonomic ranges of Hexapoda but not spiders, enabling the preferential amplification of degraded prey DNA from spider gut contents. We also developed blocking primers for further reducing off-target amplification of spider sequences and compared the performance of PCR protocols with/without the blocking primers. Based on Illumina sequencing of various families of web-weaving and non-web-weaving spider species collected in a temperate secondary forest in Japan, we examined whether multiple spider species in the community linked above- and below-ground food webs. Overall, this study shows a high-throughput pipeline for empirically characterizing prey communities consumed by spiders, providing opportunities for enhancing our understanding of how above- and below-ground food-web dynamics are coupled by generalist predators.

## Methods

### Primer design

While many predator diet studies based on high-throughput sequencing have investigated interactions between vertebrate predators and invertebrate prey [19, 23, 25], studies targeting interactions between phylogenetically close organisms generally require specialized molecular experimental protocols [28, 31]. In other words, because abundant predator DNA is highly likely to inhibit the detection of prey DNA in the analysis of invertebrate–invertebrate trophic interactions, it is often necessary to develop taxon-specific PCR primers of prey or blocking primers targeting predators for selective amplification of prey DNA [28]. However, the mitochondrial cytochrome c oxidase I (COI) region, which is used in most DNA barcoding analyses of spider diets, exhibits substantial variation in every one of three nucleotides (i.e., the third codons), even among species in the same insect orders, introducing taxonomic bias into prey community data [32, 33]. In contrast, nuclear ribosomal RNA (rRNA) regions are highly conserved across Hexapoda, providing opportunities for designing high-coverage primers for the amplification of the hyper variable internal transcribed spacer (ITS) regions. Indeed, the ITS2 region, which is flanked by the 5.8S and 28S regions, has been used for the DNA barcoding of diverse animal lineages [34].

To build a research platform for screening spider prey based on DNA metabarcoding of the ITS2 region, we designed PCR primers whose sequence matched nuclear 5.8S rRNA sequences of Hexapoda, but not those of Araneae. We first downloaded Hexapoda and Araneae sequences containing the 5.8S rRNA region from the NCBI Nucleotide database (http://www.ncbi.nlm.nih.gov/nuccore) using the keywords described in Additional file 1. The 5.8S rRNA gene sequences were then extracted using the perl program available at https://www.uni-oldenburg.de/ibu/systematik-evolutionsbiologie/programme/, and then incomplete sequences lacking the core positions of the 5.8S rRNA region were removed from the data. The sequences of each order were subjected to multiple alignment using the program MAFFT v7.272 [35] (Additional file 2).

**Fig. 1:**
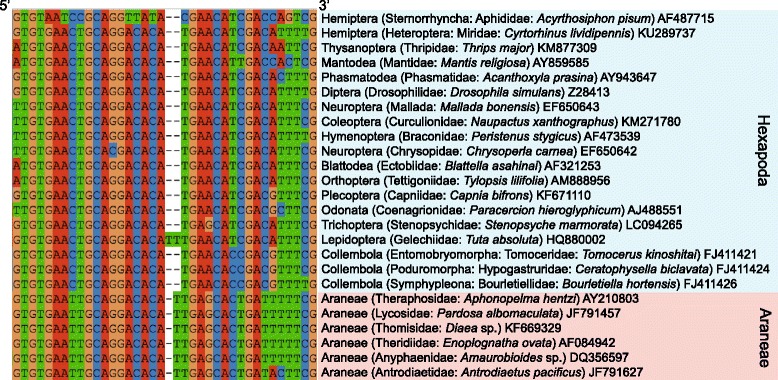
Forward primer position within 5.8S rRNA region. Around the insertion/deletion site within the 5.8S rRNA region, nucleotide sequences are well conserved within Hexapoda except for Lepidoptera and Sternorrhyncha. Hexapoda, and Araneae primers and Araneae-specific blocking primers were developed around the insertion/deletion site (Table 1)

By targeting the aligned 5.8S rRNA region, we designed a universal forward primer for most Hexapoda taxa (ITS3_Hexa_exSpF) at the highly conserved nucleotide sites (Fig. 1; Table 1; Additional file 2). As the conserved sites included an insertion/deletion between Hexapoda and Araneae at the 3′-end, the primer was expected to match preferentially to Hexapoda sequences over Araneae sequences. Of the diverse taxa examined, Lepidoptera and a group of Hemiptera (Sternorrhyncha) had unique conserved sequences around the primer position (Additional file 2). Thus, we designed additional forward primers for Lepidoptera (ITS3_Lepi_exSpF) and Sternorrhyncha (ITS3_Ster_exSpF). Note that we did not use the two primers targeting specific taxa (ITS3_Lepi_exSpF and ITS3_Ster_exSpF) in the following high-throughput sequencing analysis because we put the priority of this study on developing basic molecular experimental protocols using the primer with broader taxonomic coverage (ITS3_Hexa_exSpF). To amplify the ITS2 region, a reverse primer at the 28S rRNA region (ITS4_Hexa_R) was designed by slightly modifying an existing universal Eukaryota primers (ITS4 [36]): amplicon length was ca. 350 bp including primer positions. We also developed forward and reverse ITS2 primers for possible use in future DNA barcoding studies of Araneae (ITS3_Araneae_F and ITS3_Araneae_R).

**Table 1:** PCR primers developed in this study. Hexapoda-specific and Araneae-specific primers were developed at a 5.8S rRNA position involving deletions/insertions (Fig. 1). Reverse primers were designed at the 5′-end of 28S rRNA region. A C3 spacer was added to the 3′-end of each blocking primer for the selective amplification of Hexapoda

Category	Target	Name	Sequence (5′ – 3′)
Hexapoda primers			
Forward	Amplification of Hexapoda (excluding Lepidoptera and Sternorrhyncha)	ITS3_Hexa_exSpF	TGTGAACTGCAGGACACATGA
	Amplification of Lepidoptera	ITS3_Lepi_exSpF	TGAACTGCAGGACACATTTGA
	Amplification of Sternorrhyncha	ITS3_Ster_exSpF	CGAACATCGACMAGTCG
Reverse	Amplification of Hexapoda	ITS4_Hexa_R	TCCTCCGCTTATTAATATGC
Araneae primers			
Forward	Amplification of Araneae	ITS3_Araneae_F	TGTGAATTGCAGGACACATYG
Reverse	Amplification of Araneae	ITS4_Araneae_R	TCCTCCGCTTATTTATATGC
Blocking Primers			
Forward	Blocking of Araneae	ITS3_BlockAraneae_A	ATTGCAGGACACATTGAGC(C3)
Forward	Blocking of Araneae	ITS3_BlockAraneae_B	GACACATTGAGCACTGATT(C3)

Because high proportions of Araneae (predator) DNA could inhibit prey DNA amplification despite the use of Hexapoda-specific primers, we also designed blocking primers targeting Araneae sequences at the positions spanning the insertion/deletion (Fig. 1: Table 1). We paid special attention to include mismatches to Hexapoda sequences at or around 3′-end positions. To prevent the PCR extension of Araneae sequences, a C3 spacer [31] was added to the 3′-end of each blocking primer.

### Sampling and DNA extraction

Spiders in diverse families were sampled in a deciduous secondary forest on Mt. Yoshida, Kyoto, Japan (35°01′32′N, 135°47′10′′E) on April 12, 2016. At the study site, an evergreen oak (*Quercus glauca*) and a deciduous oak (*Quercus serrata*) were dominant, while other evergreen (e.g., *Ilex pedunculosa*) and deciduous (e.g., *Lyonia ovalifolia* and *Prunus grayana*) tree species occurred commonly. We collected spiders by beating *Q. glauca* branches at a height of 1–1.5 m with a 1-m wooden stick (2 cm in diameter) above an insect net (60 cm in diameter). All spiders > 2 mm in body length were collected individually in 2-mL microtubes (132-620C; WATSON BIO LAB) or 5-mL mailing tubes (LC3811–800; Labcon), immediately placed in a cool box. The samples were stored at − 20 °C in the laboratory.

The taxonomy of the spider community at the study site is well resolved [37], and hence all spiders were identified to species using morphology. The 235 samples representing 26 species (Additional file 3) were dissected under a stereoscopic microscope (Leica M205-C) in the laboratory. For samples of body size > 5 mm, abdomens were dissected and ground before DNA extraction: for smaller samples, the whole bodies were ground. The samples were then subjected to DNA extraction using the prepGEM Insect kit (ZyGEM). The prepGEM DNA extraction was performed with the 40-μL scale as instructed by the manufacturer and 15 μL of PCR-grade water was subsequently added to each sample tube.

### PCR and Illumina sequencing

The ITS2 region of Hexapoda was amplified using the primer pair of ITS3_Hexa_exSpF and ITS4_Hexa_R (Table 1). A two-step PCR protocol [38] was used to analyze multiple samples in a single Illumina MiSeq sequencing. In the first PCR, we used fusion primers containing Illumina sequencing primer regions, 3–6-mer Ns for improved Illumina sequencing quality [39], and specific primers (forward, 5′- TCG TCG GCA GCG TCA GAT GTG TAT AAG AGA CAG - [3–6-mer Ns] - [ITS3_Hexa_exSpF] -3′; reverse, 5′- GTC TCG TGG GCT CGG AGA TGT GTA TAA GAG ACA G - [3–6-mer Ns] - [ITS4_Hexa_R] -3′) (Additional file 4). The 10-μL reaction mixture contained 1 × KOD FX Neo buffer (TOYOBO), 0.4 mM of each dNTP, 0.2 μM of each fusion primer, 0.2 U of KOD FX Neo Polymerase (TOYOBO), and 1 μL of template DNA. To evaluate the effects of newly designed blocking primers, four types of PCR settings (blocking primer settings) were applied to the first PCR step. Specifically, ITS3_BlockAraneae_A (blocking primer A, 0.5 μM in the reaction mixture), ITS3_BlockAraneae_B (blocking primer B, 0.5 μM), both blocking primers (blocking primers A & B, 0.5 μM each), or no blocking primers was/were added to the reaction mixture. Each of the four PCR reactions were performed with a temperature profile of 94 °C for 2 min, followed by 40 cycles at 98 °C for 10 s, 50 °C for 30 s, 68 °C for 50 s, and a final extension at 68 °C for 5 min. The ramp rate was set to 1 °C/s to prevent the generation of chimeric sequences [40]. The PCR products were cleaned using 1/20 × ExoSAP-IT (affymetrix).

To add Illumina sequencing adaptors to the amplified ITS2 sequences, the second PCR reaction was performed with fusion primers containing P5/P7 Illumina adaptors, 8-mer index sequences for sample identification [41] (see Additional file 4 for forward and reverse index sequences), and partial sequencing primer sequences that bind to the 5′-end of the 1st-PCR amplicons (forward, 5′- AAT GAT ACG GCG ACC ACC GAG ATC TAC AC - [8-mer index] - TCG TCG GCA GCG TC -3′: reverse, 5′- CAA GCA GAA GAC GGC ATA CGA GAT - [8-mer index] - GTC TCG TGG GCT CGG -3′). Different sets of indexes were used for the four blocking primer experiments (Additional file 4). The temperature profile was 94 °C for 2 min, followed by 8 cycles at 98 °C for 10 s, 55 °C for 30 s, 68 °C for 50 s, and a final extension at 68 °C for 5 min (ramp rate = 1 °C/s). The PCR products were purified with AMPure XP Kit (Beckman Coulter): to remove primer dimers (< 200 bp sequences) the ratio of AMPure reagent to sample was set to 0.6 (*v*/v). For each of the four PCR (blocking primer) experimental settings, the purified PCR products of all samples were pooled. To remove remaining fusion primer dimers, an additional AMpure purification was performed for each of the four libraries. Equal concentrations of the four libraries were then mixed: note that forward-reverse index pairs differed among the four libraries to discriminate both blocking primer settings and sample numbers (Additional file 4). The pooled libraries were sequenced in a single run of the Illumina MiSeq sequencer of Graduate School of Human and Environmental Studies, Kyoto University (KYOTO-HE) (2 × 300 cycle sequencing kit; 7 pM sample concentration; 15% PhiX spike-in).

### Bioinformatics

The MiSeq Reporter program does not eliminate sequencing reads with low quality index sequences and tolerates mismatches between input and output index sequences. We thus did not use FASTQ files provided by the MiSeq sequencer, but rather converted the raw binary base call (BCL) data into FASTQ data by ourselves using the bcl2fastq v1.8.4 program distributed by Illumina, and then demultiplexed the FASTQ sequences using the program Claident v0.2.2016.07.05 [42]. All sequencing reads containing low-quality (quality score < 30) index sequences were discarded, and no mismatches between input and output index sequences were tolerated. For the remaining forward reads [data deposition: DNA DataBank of Japan (DDBJ) BioProject, PRJDB5193], filtering of reads was performed using Claident. Specifically, low-quality nucleotides were trimmed from the 3′-end until the successive five nucleotides had 30 or higher quality scores. Sequencing reads less than 170 bp in length (see [34] for length variation of the ITS2 region) and those containing certain proportions (10% or higher) of low quality (< 30) nucleotides were also eliminated. As the quality of reverse Illumina sequences is generally much lower than that of forward sequences, only forward sequences were used in the following steps. Through the stringent criteria mentioned above, 5,049,150 of 10,126,283 demultiplexed reads were discarded in total. Noisy reads were subsequently removed based on the approach proposed previously [43]. Reads that passed the filtering processes were clustered with a cut-off sequence similarity of 97% using VSEARCH [44] as implemented in Claident. Operational taxonomic units (OTUs) representing ten or less reads were then removed as OTUs representing small numbers of reads are likely to be artifacts generated through PCR/sequencing procedures.

The remaining OTUs were subjected to molecular taxonomic identification based on the database search algorithm of the query-centric auto-*k*-nearest neighbor (QCauto) methods [42] and subsequent taxonomic assignment with the lowest common ancestor (LCA) method [45] using Claident. Although we tried to remove chimeric sequences using the program UCHIME [46] with reference and *de-novo* options, not only possibly chimeric sequences but also sequences with high Blast E-scores were discarded as chimeras. Therefore, instead of using UCHIME, we simply discarded OTUs other than Hexapoda and Araneae (73.8% of the 2451 OTUs) after the QCauto–LCA taxonomic identification; even the superkingdom level taxonomic information was unavailable for the possible chimeras. In total, 50 Hexapoda and 575 Araneae OTUs were obtained (Additional file 5). The UNIX commands used in the above bioinformatics pipeline are provided in Additional file 4. No Hexapoda sequencing reads were obtained from DNA-extraction and PCR negative control samples (eight and two samples, respectively) included in the MiSeq run, while a small number of Araneae reads (0.006%) were detected from the DNA-extraction negative control samples.

The OTU count data matrix output by Claident was separated into four matrices representing respective PCR settings (Additional file 6). A cell in the matrices depicted the read count of each OTU (column) in each sample (row) (hereafter, sample-level matrices). For each matrix, relationships between the number of sequencing reads and that of Hexapoda/Araneae OTUs per sample were shown using the “rarecurve” function of the vegan v.2.4–0 package [47] of R v.3.3.1. We did not equalize the number of sequencing reads per sample by sub-sampling (cf. [48]) and used raw (i.e., non-equalized) sample-level matrices.

### Food web

The number and the taxonomic composition of detected Hexapoda OTUs across the four PCR protocols were analyzed based on the sample-level data matrices (Additional file 6). To visualize food-web structure based on the data of each PCR condition, we prepared a matrix in which rows represent an Araneae OTU, columns depict a Hexapoda OTU, and a cell entries show the number of samples from which an Araneae–Hexapoda OTU combination was observed (hereafter, Araneae × Hexapoda matrix) (Additional file 7). Based on the Araneae × Hexapoda matrix for each blocking-primer experiment, a network depicting potential trophic interactions between spiders and Hexapoda was visualized with the “plotweb” function of the R bipartite 2.3–2 package [49]. By combining the information obtained from the four blocking-primer settings, we also showed an additional food web depicting all the Araneae–Hexapoda associations detected in this study (Additional file 7).

## Results

### Effects of blocking primers

**Fig. 2:**
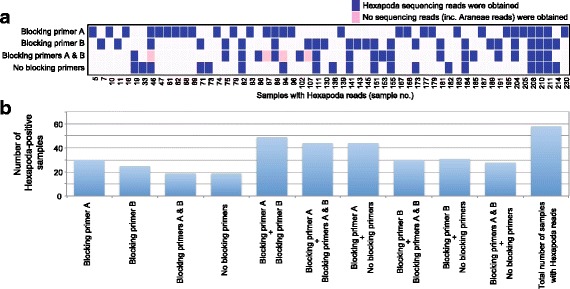
PCR settings and number of Hexapoda-positive samples. **a** Consistency/inconsistency in the detection of Hexapoda reads among the four PCR settings. The 58 samples from which Hexapoda reads were obtained in any of the four settings are shown. **b** Comparison of the number of Hexapoda-positive samples. In addition to the results from each single PCR condition, those based on the combination of two PCR settings are shown

Sequencing data were obtained from 210 individuals representing 26 spider species (Additional file 3). In all the four PCR settings, the majority of the sequencing reads obtained represented Araneae (Table 2). The percentage of Hexapoda reads compared to total arthropod reads were 1.08, 1.46, 0.65, and 0.90 for the blocking primer A, blocking primer B, blocking primers A & B, and no blocking primer settings, respectively (Table 2). The percentage of samples with Hexapoda reads also varied among the PCR settings, ranging from 9.2% (no blocking primer) to 15.0% (blocking primer A). At the maximum, six Hexapoda OTUs were detected from a sample (Additional file 8a-d). On average, 3428–3985 Araneae reads and 258–445 Hexapoda reads were obtained from each sample depending on the PCR settings (Table 2). The number of Araneae OTUs obtained per sample varied considerably among spider species (Additional files 8e-h and 9), suggesting interspecific variation in the number of rRNA tandem repeats and/or the level of intragenomic ITS variation. When data from all four PCR settings were combined, the percentage of Hexapoda-positive samples was 27.6% (58/210; Table 2; Fig. 2).

**Table 2:** Summary of Illumina sequencing. Results based on the four PCR (blocking primer) settings are separately shown

Experiment	No. of Samples with reads	No. of Samples with Hexapoda reads	Samples with Hexapoda reads (%)	Mean no. reads per sample (Hexapoda)	Mean no. reads per sample (Araneae)	Total no. of reads (Hexapoda)	Total no. of reads (Araneae)	Percentage of Hexapoda reads (%)
Blocking primer A	200	30	15.0	271	3754	8130	750,862	1.08
Blocking primer B	204	25	12.3	445	3720	11,113	758,851	1.46
Blocking primers A & B	189	19	10.1	258	3985	4909	753,181	0.65
No blocking primers	207	19	9.2	335	3428	6363	709,647	0.90
Total	210	58	27.6	–	–	30,515	2,972,541	1.03

### Community composition of detected Hexapoda

**Fig. 3:**
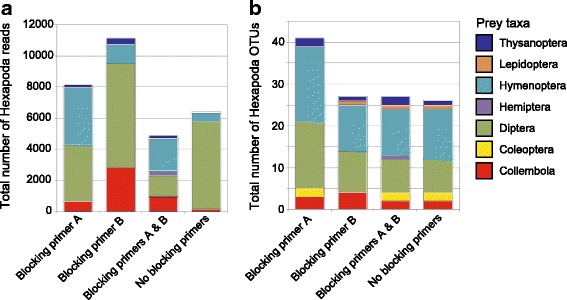
Taxonomic composition of obtained Hexapoda reads and OTUs. **a** Taxonomic composition of Hexapoda sequencing reads in the four PCR settings. **b** Taxonomic composition of Hexapoda OTUs in the four PCR settings

The high-throughput sequencing data revealed reads assigned to Collembola and six insect orders (Coleoptera, Diptera, Hemiptera, Hymenoptera, Lepidoptera, and Thysanoptera) (Fig. 3; Additional file 6). Although we expected the Hexapoda-specific primer to mismatch sequences of Lepidoptera [and a group of Hemiptera (Sternorrhyncha)], a Lepidoptera OTU was detected in three of the four PCR settings (Fig. 3). The Hemiptera OTU observed represented Auchenorrhyncha, but not Sternorrhyncha (Additional file 6). The composition of Hexapoda taxa varied slightly across the four PCR settings. The number of Hexapoda OTUs observed was the highest in the blocking primer A condition (Fig. 3).

### Araneae–Hexapoda food web

**Fig. 4:**
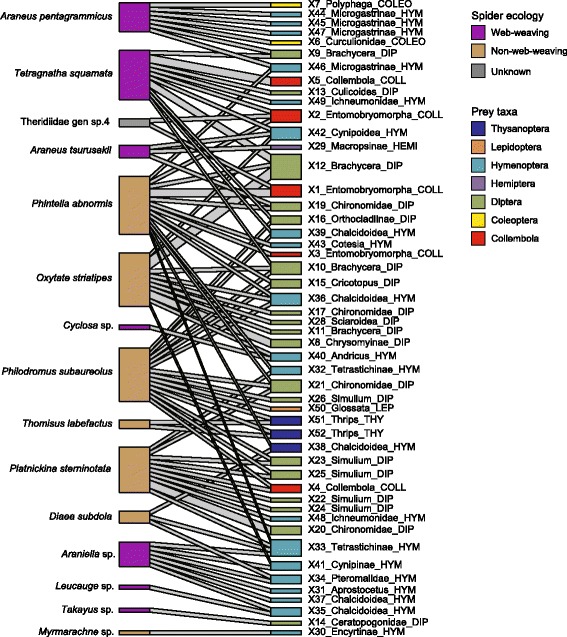
Food-web structure. Spider species (left) are linked to the Hexapoda OTUs (right) detected. The results of all the four PCR settings were combined. Web-weaving and non-web-weaving spiders are indicated by color. The thickness of the link represents the number of spider samples from which a focal spider–Hexapoda association was observed. The lowest taxonomic rank indicated by the automatic molecular identification is shown for each prey OTU, followed by the abbreviation of order-level taxonomy (Additional file 6). Box size represents the number of samples. COLL, Collembola; COLEO, Coleoptera; DIP, Diptera; HEMI, Hemiptera; HYM, Hymenoptera; LEPI, Lepidoptera; THY, Thysanoptera

By combining all the Araneae–Hexapoda associations revealed in this study (Additional file 10), a network involving 15 spider species and 50 insect/springtail OTUs was obtained (Fig. 4); note that intragenomic variation of ITS sequences might, in general, result in the overestimation of taxa or species. The network suggests that three spider species (*Tetragnatha squamata*, *Phintella abnormis*, and *Platnickina sterninotata*) at the study site prey upon both insects and springtails. Even a species whose prey is thought to consist primarily of spiders (*P. sterninotata*) [50] potentially preyed on springtails, Hymenoptera, and Diptera species. Many other spider species were inferred to prey upon various above-ground insects, partly sharing prey taxa. In the network, there were Hymenoptera OTUs belonging to families consisting mainly of parasitoids (Braconidae, Pteromalidae, Encyrtidae, Eulophidae, and Ichneumonidae) and the family of gall wasps (Cynipidae) (Fig. 4: Additional file 6).

## Discussion

The results implied that multiple spider species in a temperate forest preyed not only on insects but also on springtails. Thus, while pioneering studies have focused on the diets of a single or a few spider species [8, 9, 11, 13, 18], this study provided a novel platform for evaluating how spider communities as a whole drive ecosystem-scale processes. Our DNA metabarcoding data also suggested that spiders eat diverse taxonomic groups of insects, potentially having impacts on populations of various prey species in food webs [51]. Meanwhile, prey taxonomic compositions varied, to some extent, among spider species in the secondary forest (Fig. 4), although the number of samples per species needs to be increased in future studies to perform statistical tests of niche (prey) overlap.

The results also suggested that spiders interact with multiple trophic levels of Hexapoda species in food webs. Specifically, the network revealed in this study (Fig. 4) involved not only herbivorous and fungivorous (or detritivorous) lineages of Hexapoda but also parasitoids in the order Hymenoptera. Among the parasitic Hymenoptera families detected from the spider samples, only Ichneumonidae includes well-characterized species known to parasitize on spiders [52, 53], while others (Braconidae, Pteromalidae, Encyrtidae, and Eulophidae) consist mainly of parasitoids on insects or species with unknown life histories [54, 55]. Because our spider samples did not seem to carry eggs/larvae/pupae of spider-specific Ichneumonidae wasps, which are easily recognizable ectoparasites [52, 53], most parasitoid hymenopterans detected in this study may be prey of the examined spider samples. However, a DNA metabarcoding analysis alone does not indicate whether such parasitoids are directly preyed on by spiders or they were just parasitizing on Hexapoda hosts caught by spiders. In either case, interactions between spiders and parasitoid wasps are of particular interest, as predation/parasitism within a trophic level has been considered as a strong determinant of food-web dynamics [56–59]. Moreover, existing DNA barcoding methods, including ours, are not designed to distinguish between signs of cannibalism, intra-guild predation, scavenging, and secondary predation (i.e., DNA of prey’s prey). In addition, with the present DNA-metabarcoding protocol, we may occasionally detect arthropod DNA contaminated in the environment. Further methodological improvements such as individual-level genotyping (e.g., single nucleotide polymorphism) for intra-guild predation analyses and sodium-hypochlorite bleaching for degrading non-prey DNA on predator sample surfaces will allow more comprehensive research of food-web structure.

Although supporting evidences based on direct field observations of predation events are important for correctly evaluating trophic interactions, DNA metabarcoding-based analyses are expected to provide insights into the underappreciated structure of food webs [20, 48, 60]. For example, our data suggest trophic interactions between insects and *P. sterninotata*, which had been recognized as spider-eating species. Although the present DNA-based analysis might have detected insects preyed on by spider prey of *P. sterninotata* (i.e., secondary predation), DNA metabarcoding studies potentially help identify signs of novel trophic interactions in the wild (sensu [20]). In addition, DNA metabarcoding is a powerful tool for systematically investigating prey compositions of a whole spider community because many spider species are nocturnal [61–63], making their predation behavior—especially that of small-sized species—difficult to observe directly in the field,.

The high-throughput sequencing technology outlined in this study is expected to accelerate studies examining how spiders play key ecosystem roles at the interface of above- and below-ground biological communities. Using the protocol described in this study, molecular experimental procedures from DNA extraction to sequencing-library preparation could be completed in a few days, even with hundreds of spider samples. Illumina MiSeq sequencing and bioinformatics procedures also take only a few additional days. Therefore, the molecular experimental and bioinformatics pipeline allowing fast profiling of predator–prey interactions is expected to provide an avenue for understanding temporal dynamics of food webs and their ecosystem-level consequences [64]. In particular, we will be able to examine how phenological switching between above- and below-ground prey by spiders can promote/inhibit the coexistence of species at the lower trophic levels [65]. The “early season predation” hypothesis predicts that the high biomass of overwintering adult/juvenile spiders is sustained by alternative prey, such as detritivores, in early spring and that the resultant initial asymmetry in predator and above-ground insect biomass (spiders > insects) can restrict outbreaks of herbivores in the successive foliation season [66]. Understanding of this phenological mechanism of predator–prey interactions, therefore, is invaluable from the aspect of insect pest controls in the restoration of natural ecosystems and the management of farmlands [15, 67]. By applying the high-throughput methods described herein to time-series sample sets, the early season predation hypothesis can be tested.

To enable a more comprehensive understanding of linkages between above- and below-ground food webs, the protocol proposed in this study needs further optimization and improvements. The comparative analysis of four PCR settings (Fig. 2 and Additional file 10) suggested that the use of blocking primers increased the number of the spider–Hexapoda associations detected, while prey information was available to some extent even without blocking primers as suggested in a previous study [27]. We also found that the four PCR settings varied slightly in observed prey taxonomic compositions (Fig. 3; Additional file 10). To avoid the loss of information, it may be important to combine results based on at least two PCR settings; combining the results of the blocking primer A and B settings (but not the simultaneous use of the two primers) may be the most informative (Fig. 2b). Alternatively, we may be able to improve the binding efficiency of the blocking primers by synthesizing them as peptide nucleic acid (PNA) [26]. Meanwhile, given that Hexapoda sequences were not obtained from many of our samples (Table 2), improvements in DNA extraction or PCR protocols may further enhance prey information. Enrichment of fragmented prey DNA in template DNA solution, for example, has been reported to increase prey DNA detection rates [28] (see also [68]). In addition, by increasing the concentration of blocking primers in PCR reaction mixtures, more selective amplification of Hexapoda sequences over spider sequences may result in more enriched prey detection.

In this study, we developed a DNA barcoding method targeting the nuclear ITS2 region in order to overcome the previously indicated shortcomings of the use of the mitochondrial COI region [32, 33]. Most importantly, due to the lack of highly conserved regions within the COI region, COI primers that have been commonly used in DNA barcoding studies [69–71] often mismatch the template DNA of arthropods, introducing taxonomic bias into amplicon libraries [32, 33]. In contrast, nucleotide sequences of the rRNA genes flanking the ITS2 region are highly conserved (Fig. 1), drastically reducing mismatches between primers and template genomic DNA and thereby increasing taxonomic coverage. Moreover, as the rRNA regions do not encode proteins, we could use insertion/deletion sites for developing taxon-specific (Araneae-specific) blocking primers (Fig. 1; Table 1). Despite the benefits of using the ITS2 region, the number of ITS sequences has been much smaller than that of COI sequences in public databases. As a result, most prey species in the inferred food web were unidentified even at the genus or family level, making it difficult to confirm that the detected Collembola sequences derived from below-ground fungivore/detritivore taxa or springtail species inhabiting canopies or living-tree bark. The paucity of ITS sequences in public databases reflects the history of DNA barcoding: during the era of Sanger sequencing, organelle (i.e., haploid) markers including mitochondrial COI had advantage over nuclear markers because the latter required elaborate cloning processes to obtain clear electropherograms (see [72] for additional merits of COI as a marker). This situation is changing rapidly as a single run of a next-generation sequencer now enables the generation of nuclear marker databases of hundreds or thousands of samples [48]. Indeed, an increasing number of researchers use the ITS regions for the DNA barcoding of not only fungi [73] but also animals [34] and plants [74], enhancing public ITS sequence databases. Developing ITS sequence databases of model local communities is also encouraged in order to understand how diverse arthropod guilds structure food webs.

Despite the use of Hexapoda-specific primers and Araneae-specific blocking primers, the majority of sequencing reads obtained represented spiders rather than their Hexapoda prey (Table 2). While further methodological improvements will increase the proportion of prey reads as discussed above, spider reads per se may provide important information. For example, given that juveniles of small spider species are often indistinguishable, DNA barcoding information can help identification of not only prey but also predators. When we use the ITS2 region for DNA barcoding, however, we need to take into account potential intragenomic sequence variation of the region [34, 75]. Our data suggested that most spider species analyzed in this study had two or more intragenomic ITS2 variants (Additional files 8 2e–h and 9). Such intragenomic variation can promote, rather than prevent, the use of the ITS2 region for molecular taxonomic identification. In theory, two or more sequence variants per species need to be deposited to public databases for reliable taxonomic assignment of a query sequence at the species level [42]. Therefore, database construction of organelle markers essentially requires two or more reference samples per species, while the number of reference samples can be reduced in DNA barcoding based on the ITS regions in the presence of intragenomic variation. The fact that nuclear markers are almost free from misidentification due to past introgressive hybridization [76] is another reason for promoting nuclear-marker-based DNA barcoding. We hope the primers developed in this study (Table 1) help accelerate ITS-based DNA barcoding, whose benefits have remained underappreciated in taxonomic and ecological studies of arthropods.

## Conclusions

In the present study, we developed a DNA metabarcoding method for analyzing food webs involving both below-ground and above-ground arthropods. The results support the working hypothesis that multiple spider species in the study community prey on both below- and above-ground prey. This study also suggests that parasitoid wasps are important components of the diets of spiders, illuminating the structure of an arthropod food web involving various trophic levels. Although COI markers have huge merits in terms of the richness of reference database information, further improvements in ITS-marker-based protocols (e.g., use of PNA blocking primers) will provide prospective technical options for testing hypotheses on the coupling of above- and below-ground ecosystem processes. More case studies are needed to better understand how springtails and other below-ground fungivores and detritivores [23] [e.g., mites (Acari) and fungus gnats (Sciaridae)] are involved in the entire food webs of terrestrial ecosystems.

## Additional files

Additional file 1: **Data S1.** Keywords used in the downloading of ITS sequences in the NCBI database. (TXT 1 kb)

Additional file 2: **Data S2.** Aligned 5.8S rRNA database sequences. (TXT 387 kb)

Additional file 3: **Table S1.** List of spider samples. (PDF 72 kb)

Additional file 4: **Data S3.** Fusion primers, index tags, and the UNIX codes used in the Illumina sequencing and bioinformatic procedures. (XLSX 211 kb)

Additional file 5: **Data S4.** Hexapoda and Araneae OTU sequences. (TXT 158 kb)

Additional file 6: **Data S5.** Sample-level matrices and taxonomy of OTUs. (XLSX 363 kb)

Additional file 7: **Data S6.** Araneae × Hexapoda matrices. (XLSX 46 kb)

Additional file 8: **Figure S1.** Rarefaction curves of the sequencing reads. Each curve represents relationship between the number of sequencing reads and the number of observed OTUs. (PDF 522 kb)

Additional file 9: **Table S2.** Summary of Araneae ITS sequence variants detected. (PDF 78 kb)

Additional file 10: **Figure S2.** Spider–Hexapoda networks revealed in each PCR condition. (PDF 634 kb)
