# Supplementary material for: DNA metabarcoding of spiders, insects, and springtails for exploring potential linkage between above- and below-ground food webs
Source: Zoological Lett. 2018 Feb 15;4:4. doi: 10.1186/s40851-018-0088-9 (PMC5815251; doi:10.1186/s40851-018-0088-9)
Supplement: Supplementary file 3 — Table S1. List of spider samples. (PDF 72 kb) [file 40851_2018_88_MOESM3_ESM.pdf]

Additional file 3: Table S1. List of spider samples.

Species	Family	Ecology	*N*
Araneus pentagrammicus Karsch, 1879	Araneidae	Web-weaving	3
Araneus tsurusakii Tanikawa, 2001	Araneidae	Web-weaving	2
Araniella sp.	Araneidae	Web-weaving	2
Araniella yaginumai Tanikawa, 1995	Araneidae	Web-weaving	1
Cyclosa sp.	Araneidae	Web-weaving	1
Neolinyphia sp.	Linyphiidae	Web-weaving	1
Oxyopes sertatus L. Koch, 1878	Oxyopidae	Non-web-weaving	2
Philodromus subaureolus Bösenberg & Strand 1906	Philodromidae	Non-web-weaving	44
Myrmarachne sp.	Salticidae	Non-web-weaving	3
Phintella abnormis (Bösenberg & Strand 1906)	Salticidae	Non-web-weaving	42
Pseudicius kimjoopili	Salticidae	Non-web-weaving	1
Leucauge sp.	Tetragnathidae	Web-weaving	1
Tetragnatha squamata Karsch, 1879	Tetragnathidae	Web-weaving	42
Chrysso foliata (L. Koch, 1878)	Theridiidae	Web-weaving	2
Dipoena punctisparsa Yaginuma, 1967	Theridiidae	Web-weaving	1
Platnickina sterninotata (Bösenberg et Strand, 1906)	Theridiidae	Non-web-weaving	27
Takayus sp.	Theridiidae	Web-weaving	1
Theridiidae gen sp.1	Theridiidae	Unknown	1
Theridiidae gen sp.2	Theridiidae	Unknown	1
Theridiidae gen sp.3	Theridiidae	Unknown	1
Theridiidae gen sp.4	Theridiidae	Unknown	1
Diaea subdola O. Pickard-Cambridge, 1885	Thomisidae	Non-web-weaving	9
Ebelingia kumadai (Ono, 1985)	Thomisidae	Non-web-weaving	1
Lystiteles sp.	Thomisidae	Non-web-weaving	2
Oxytate striatipes L. Koch, 1878	Thomisidae	Non-web-weaving	17
Thomisus labefactus Karsch, 1881	Thomisidae	Non-web-weaving	1
