# Supplementary material for: DNA metabarcoding of spiders, insects, and springtails for exploring potential linkage between above- and below-ground food webs
Source: Zoological Lett. 2018 Feb 15;4:4. doi: 10.1186/s40851-018-0088-9 (PMC5815251; doi:10.1186/s40851-018-0088-9)
Supplement: Supplementary file 8 — Figure S1. Rarefaction curves of the sequencing reads. Each curve represents relationship between the number of sequencing reads and the number of observed OTUs. (PDF 522 kb) [file 40851_2018_88_MOESM8_ESM.pdf]

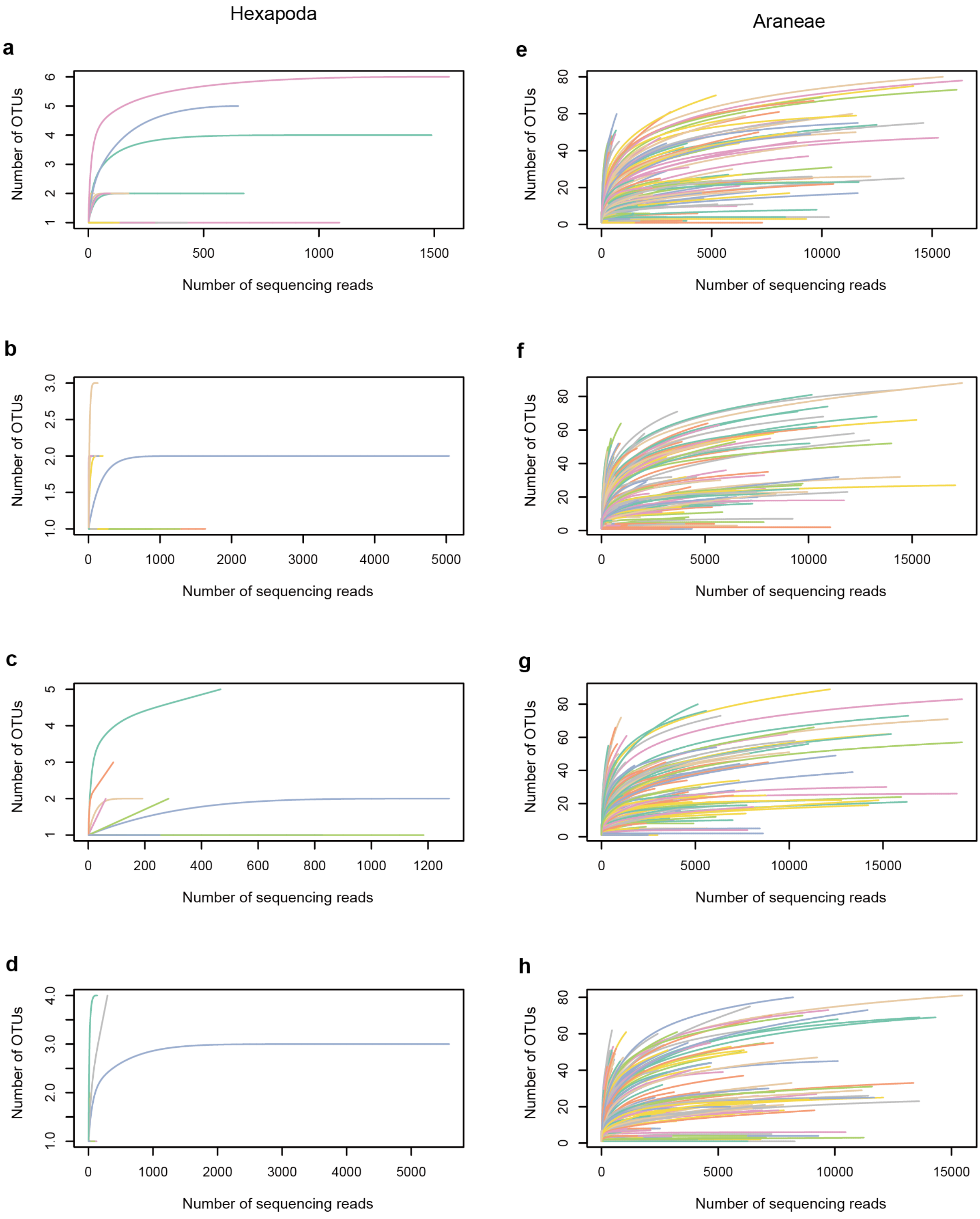

Additional file 8: Figure S1. Rarefaction curves of the sequencing reads. Each curve represents relationship between the number of sequencing reads and the number of observed OTUs (left, Hexapoda read data; right, Araneae read data). **a**, **e** Blocking primer A condition. **b**, **f** Blocking primer B condition. **c**, **g** Blocking primers A & B condition. **d**, **h** No blocking primer condition.
