# Supplementary material for: DNA metabarcoding of spiders, insects, and springtails for exploring potential linkage between above- and below-ground food webs
Source: Zoological Lett. 2018 Feb 15;4:4. doi: 10.1186/s40851-018-0088-9 (PMC5815251; doi:10.1186/s40851-018-0088-9)
Supplement: Supplementary file 9 — Table S2. Summary of Araneae ITS sequence variants detected. (PDF 78 kb) [file 40851_2018_88_MOESM9_ESM.pdf]

Additional file: Table S2. Summary of Araneae ITS sequence variants detected.

(A) Mean number of Araneae OTUs per sample.

Species	Family	Blocking primer A	Blocking primer B	Blocking primers A & B	No blocking primers
Araneus pentagrammicus	Araneidae	2.5	15.7	24.5	16.7
Araneus tsurusakii	Araneidae	1.5	1.5	1.5	1.5
Araniella sp.	Araneidae	6.0	23.0	19.0	23.0
Araniella yaginumai	Araneidae	6.0	2.0	n.a.	2.0
Cyclosa sp.	Araneidae	4.0	14.0	15.0	15.0
Neolinyphia sp.	Linyphiidae	2.0	2.0	n.a.	4.0
Oxyopes sertatus	Oxyopidae	1.5	26.5	26.0	22.0
Philodromus subaureolus	Philodromidae	18.0	26.9	25.7	27.6
Myrmarachne sp.	Salticidae	22.0	24.3	22.0	20.7
Phintella abnormis	Salticidae	52.2	29.4	28.7	28.5
Pseudicius kimjoopili	Salticidae	1.0	1.0	1.0	1.0
Leucauge sp.	Tetragnathidae	1.0	5.0	n.a.	6.0
Tetragnatha squamata	Tetragnathidae	35.0	30.7	31.7	31.0
Chrysso foliata	Theridiidae	1.0	27.0	12.5	22.5
Dipoena punctisparsa	Theridiidae	8.0	7.0	5.0	8.0
Platnickina sterninotata	Theridiidae	5.0	21.9	21.1	21.7
Takayus sp.	Theridiidae	1.0	1.0	1.0	1.0
Theridiidae gen sp.1	Theridiidae	n.a.	22.0	20.0	17.0
Theridiidae gen sp.2	Theridiidae	2.0	2.0	2.0	3.0
Theridiidae gen sp.3	Theridiidae	4.0	61.0	50.0	54.0
Theridiidae gen sp.4	Theridiidae	6.0	46.0	57.0	53.0
Diaea subdola	Thomisidae	1.4	24.4	21.1	25.4
Ebelingia kumadai	Thomisidae	2.0	2.0	2.0	1.0
Lystiteles sp.	Thomisidae	3.5	43.5	41.5	56.5
Oxytate striatipes	Thomisidae	21.4	24.0	25.2	22.8
Thomisus labefactus	Thomisidae	4.0	18.0	12.0	12.0

(B) Mean number of Araneae OTUs per sample [(no. of OTUs)/(1000 Araneae reads)].

Species	Family	Blocking primer A	Blocking primer B	Blocking primers A & B	No blocking primers
Araneus pentagrammicus	Araneidae	6.0	42.5	23.0	18.1
Araneus tsurusakii	Araneidae	0.6	0.8	0.5	0.6
Araniella sp.	Araneidae	2.1	46.4	100.2	56.3
Araniella yaginumai	Araneidae	6.4	2.3	n.a.	1.2
Cyclosa sp.	Araneidae	0.5	3.6	14.9	15.5
Neolinyphia sp.	Linyphiidae	1.2	14.6	n.a.	3.6
Oxyopes sertatus	Oxyopidae	1.0	6.2	8.9	5.0
Philodromus subaureolus	Philodromidae	5.2	41.3	60.7	34.9
Myrmarachne sp.	Salticidae	4.3	2.9	2.7	12.0
Phintella abnormis	Salticidae	11.4	21.4	32.3	26.4
Pseudicius kimjoopili	Salticidae	0.1	25.6	0.6	0.2
Leucauge sp.	Tetragnathidae	16.1	10.4	n.a.	7.2
Tetragnatha squamata	Tetragnathidae	128.8	56.1	64.1	58.9
Chrysso foliata	Theridiidae	0.7	4.2	5.6	4.9
Dipoena punctisparsa	Theridiidae	0.8	0.8	0.9	3.2
Platnickina sterninotata	Theridiidae	23.0	58.7	52.4	47.2
Takayus sp.	Theridiidae	0.7	0.3	0.4	13.3
Theridiidae gen sp.1	Theridiidae	n.a.	6.0	3.3	6.2
Theridiidae gen sp.2	Theridiidae	1.4	2.3	2.9	1.7
Theridiidae gen sp.3	Theridiidae	6.6	9.7	7.8	20.1
Theridiidae gen sp.4	Theridiidae	3.9	19.9	5.3	9.6
Diaea subdola	Thomisidae	2.5	21.3	25.1	16.8
Ebelingia kumadai	Thomisidae	0.5	2.6	1.1	0.9
Lystiteles sp.	Thomisidae	0.8	88.5	70.9	55.9
Oxytate striatipes	Thomisidae	9.6	16.4	16.7	31.3
Thomisus labefactus	Thomisidae	0.4	2.5	2.0	10.9
