# Supplementary material for: DNA metabarcoding of spiders, insects, and springtails for exploring potential linkage between above- and below-ground food webs
Source: Zoological Lett. 2018 Feb 15;4:4. doi: 10.1186/s40851-018-0088-9 (PMC5815251; doi:10.1186/s40851-018-0088-9)
Supplement: Supplementary file 10 — Figure S2. Spider–Hexapoda networks revealed in each PCR condition. (PDF 634 kb) [file 40851_2018_88_MOESM10_ESM.pdf]

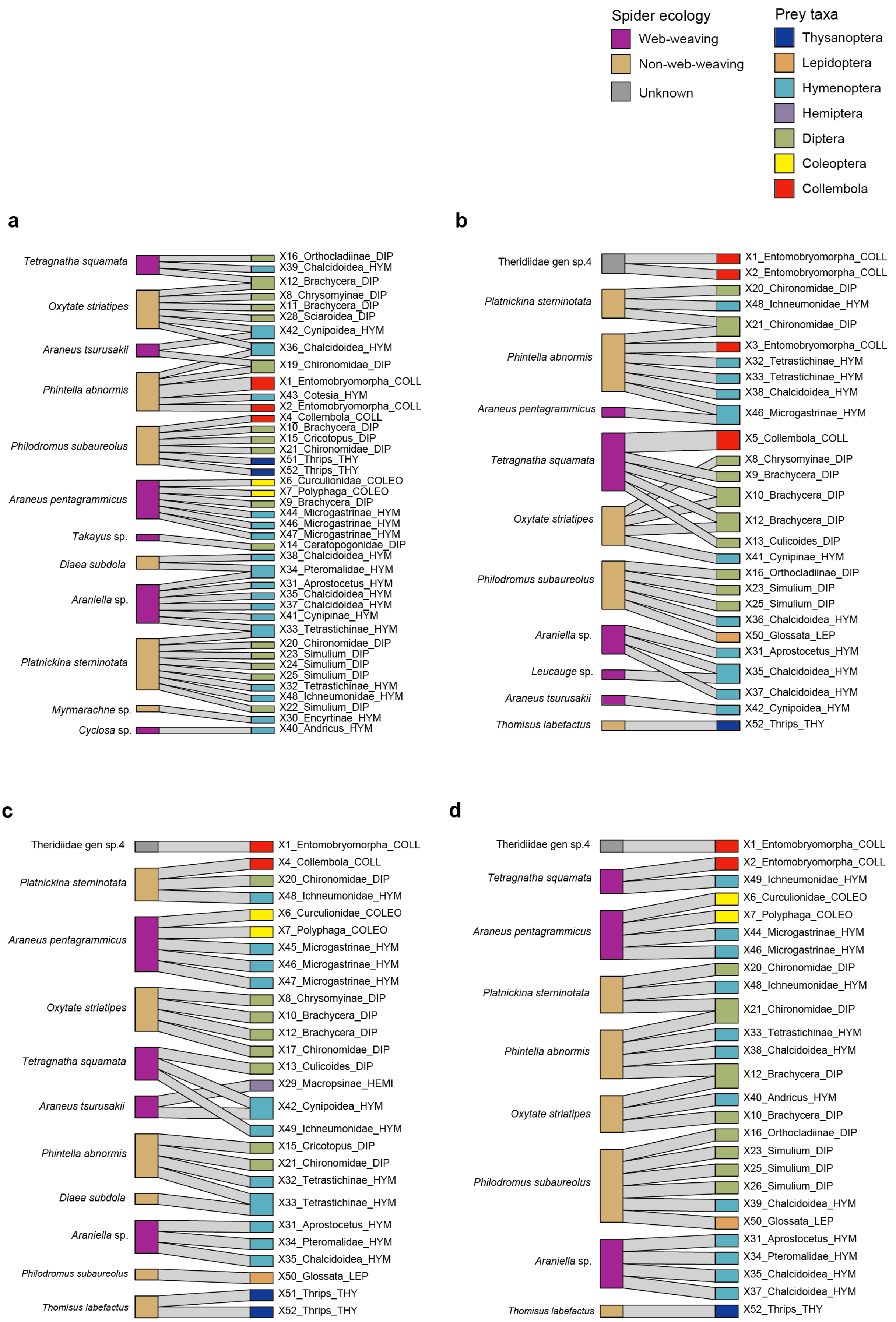
Spider ecology
Web-weaving
Non-web-weaving
Unknown
Prey taxa
Thysanoptera
Lepidoptera
Hymenoptera
Hemiptera
Diptera
Coleoptera
Collembola
a
Tetragnatha squamata
Oxytate striatipes
Araneus tsurusakii
Phintella abnormis
Philodromus subaureolus
Araneus pentagrammicus
Takayus sp.
Diaea subdola
Araniella sp.
Platnickina sterninotata
Myrmarachne sp.
Cyclosa sp.
X16_Orthocladiinae_DIP
X39_Chalcidoidea_HYM
X12_Brachycera_DIP
X8_Chrysomyinae_DIP
X11_Brachycera_DIP
X28_Sciaroidea_DIP
X42_Cynipoidea_HYM
X36_Chalcidoidea_HYM
X19_Chironomidae_DIP
X1_Entomobryomorpha_COLL
X43_Cotesia_HYM
X2_Entomobryomorpha_COLL
X4_Collembola_COLL
X10_Brachycera_DIP
X15_Cricotopus_DIP
X21_Chironomidae_DIP
X51_Thrips_THY
X52_Thrips_THY
X6_Curculionidae_COLEO
X7_Polyphaga_COLEO
X9_Brachycera_DIP
X44_Microgastrinae_HYM
X46_Microgastrinae_HYM
X47_Microgastrinae_HYM
X14_Ceratopogonidae_DIP
X38_Chalcidoidea_HYM
X34_Pteromalidae_HYM
X31_Aprostocetus_HYM
X35_Chalcidoidea_HYM
X37_Chalcidoidea_HYM
X41_Cynipinae_HYM
X33_Tetrastichinae_HYM
X20_Chironomidae_DIP
X23_Simulium_DIP
X24_Simulium_DIP
X25_Simulium_DIP
X32_Tetrastichinae_HYM
X48_Ichneumonidae_HYM
X22_Simulium_DIP
X30_Encyrtinae_HYM
X40_Andricus_HYM
b
Theridiidae gen sp.4
Platnickina sterninotata
Phintella abnormis
Araneus pentagrammicus
Tetragnatha squamata
Oxytate striatipes
Philodromus subaureolus
Araniella sp.
Leucauge sp.
Araneus tsurusakii
Thomisus labefactus
X1_Entomobryomorpha_COLL
X2_Entomobryomorpha_COLL
X20_Chironomidae_DIP
X48_Ichneumonidae_HYM
X21_Chironomidae_DIP
X3_Entomobryomorpha_COLL
X32_Tetrastichinae_HYM
X33_Tetrastichinae_HYM
X38_Chalcidoidea_HYM
X46_Microgastrinae_HYM
X5_Collembola_COLL
X8_Chrysomyinae_DIP
X9_Brachycera_DIP
X10_Brachycera_DIP
X12_Brachycera_DIP
X13_Culicoides_DIP
X41_Cynipinae_HYM
X16_Orthocladiinae_DIP
X23_Simulium_DIP
X25_Simulium_DIP
X36_Chalcidoidea_HYM
X50_Glossata_LEP
X31_Aprostocetus_HYM
X35_Chalcidoidea_HYM
X37_Chalcidoidea_HYM
X42_Cynipoidea_HYM
X52_Thrips_THY
c
Theridiidae gen sp.4
Platnickina sterninotata
Araneus pentagrammicus
Oxytate striatipes
Tetragnatha squamata
Araneus tsurusakii
Phintella abnormis
Diaea subdola
Araniella sp.
Philodromus subaureolus
Thomisus labefactus
X1_Entomobryomorpha_COLL
X4_Collembola_COLL
X20_Chironomidae_DIP
X48_Ichneumonidae_HYM
X6_Curculionidae_COLEO
X7_Polyphaga_COLEO
X45_Microgastrinae_HYM
X46_Microgastrinae_HYM
X47_Microgastrinae_HYM
X8_Chrysomyinae_DIP
X10_Brachycera_DIP
X12_Brachycera_DIP
X17_Chironomidae_DIP
X13_Culicoides_DIP
X29_Macropsinae_HEMI
X42_Cynipoidea_HYM
X49_Ichneumonidae_HYM
X15_Cricotopus_DIP
X21_Chironomidae_DIP
X32_Tetrastichinae_HYM
X33_Tetrastichinae_HYM
X31_Aprostocetus_HYM
X34_Pteromalidae_HYM
X35_Chalcidoidea_HYM
X50_Glossata_LEP
X51_Thrips_THY
X52_Thrips_THY
d
Theridiidae gen sp.4
Tetragnatha squamata
Araneus pentagrammicus
Platnickina sterninotata
Phintella abnormis
Oxytate striatipes
Philodromus subaureolus
Araniella sp.
Thomisus labefactus
X1_Entomobryomorpha_COLL
X2_Entomobryomorpha_COLL
X49_Ichneumonidae_HYM
X6_Curculionidae_COLEO
X7_Polyphaga_COLEO
X44_Microgastrinae_HYM
X46_Microgastrinae_HYM
X20_Chironomidae_DIP
X48_Ichneumonidae_HYM
X21_Chironomidae_DIP
X33_Tetrastichinae_HYM
X38_Chalcidoidea_HYM
X12_Brachycera_DIP
X40_Andricus_HYM
X10_Brachycera_DIP
X16_Orthocladiinae_DIP
X23_Simulium_DIP
X25_Simulium_DIP
X26_Simulium_DIP
X39_Chalcidoidea_HYM
X50_Glossata_LEP
X31_Aprostocetus_HYM
X34_Pteromalidae_HYM
X35_Chalcidoidea_HYM
X37_Chalcidoidea_HYM
X52_Thrips_THY

Additional file 10: Figure S2. Spider–Hexapoda networks revealed in each PCR condition. **a** Blocking primer A condition. Web-weaving and non-web-weaving spiders are indicated by color. The thickness of the link represents the number of spider samples from which a focal spider–Hexapoda association was observed. The lowest taxonomic rank indicated by the automatic molecular identification is shown for each prey OTU, followed by the abbreviation of order-level taxonomy (Supplementary Data 5). Box size represents the number of samples. COLL, Collembola; COLEO, Coleoptera; DIP, Diptera; HEMI, Hemiptera; HYM, Hymenoptera; LEPI, Lepidoptera; THY, Thysanoptera. **b** Blocking primer B condition. **c** Blocking primers A & B condition. **d** No blocking primer condition.
